# Data on rotary die filling performance of various pharmaceutical powders

**DOI:** 10.1016/j.dib.2020.106220

**Published:** 2020-08-24

**Authors:** Xue Tang, Ling Zhang, Zhen-Feng Wu, Ping Sun, Chuan-Yu Wu

**Affiliations:** a Key Laboratory of Modern Preparation of TCM, Ministry of Education, Jiangxi University of Traditional Chinese Medicine, Nanchang 330004, China; b Department of Chemical and Process Engineering, University of Surrey, Guildford GU2 7XH, United Kingdom; c Jiangzhong Pharmaceutical Co., Ltd., Nanchang 330096, China

**Keywords:** Die filling, Tabletting, Rotary press, Weight variation, Feed frame

## Abstract

As one of critical process steps during pharmaceutical tabletting, rotary die filling is still not well understood. To address this issue, a model rotary die filling system with a paddle feeder was developed to closely mimic the industrial process. Using this model system, the performance of various pharmaceutical powders at different turret and paddle speeds was evaluated, and the dependence of fill variation on process conditions and material properties was examined. A comprehensive dataset was created and reported here to show the effects of material and process parameters on the die filling performance and the filling consistency. It is believed that the data can also be used for data-driven process modelling and for developing robust machine learning models for pharmaceutical manufacturing.

**Specifications Table**

**Table:** 

**Subject**	Pharmaceutics
**Specific subject area**	Powder filling; die filling; tabletting
**Type of data**	Tables
**How data were acquired**	A model rotary die filling system with a paddle feeder was developed. The turret speed was measured and calibrated with an Advent optical tachometer (A2103/LSR, Compact Instruments, UK). The fill wight was measured using an analytical balance (Entris224–1S, Sartorius, Germany).
**Data format**	Raw and analysed data.
**Parameters for data collection**	The room temperature was below 30 °C and the relative humidity was below 50%, when collecting the data.
**Description of data collection**	The masses of 3 different powders deposited into 4 dies during die filling processes at various turret and paddle speeds were measured and recorded.
**Data source location**	University of Surrey Guildford United Kindom
**Data accessibility**	Data is supplied with this article
**Related research article**	Xue Tang, Anastasiya. Zakhvatayeva, Ling Zhang, Zhen-Feng Wu, Ping Sun, Chuan-Yu Wu, Flow behaviour of pharmaceutical powders during rotary die filling with a paddle feeder, International Journal of Pharmaceutics, Vol. 585, 2020, 119547. https://doi.org/10.1016/j.ijpharm.2020.119547

**Value of the Data**

• The data provides evidence supporting the correlation between the weight variation and powder flowability
• Researchers and scientists working on pharmaceutical process and formulation development can use the data to further examine the impact of powder flow behaviour on the quality of the tablets produced using rotary tabletting presses. The data can also be used to guide the formulation design to minimise the tablet weight variation.
• Computational modellers can also use the data to develop computational intelligence models for pharmaceutical manufacturing.

## 1 Data Description

A total of 614 experiments were performed, which included three repetitions for each test. For each experiment, the mass of deposited powder was measured. The average value, standard deviation and coefficient of variation (CV) for the fill ratios of three different materials were then calculated. These analysed data are presented using three tables for each material: Tables 1–3 for MCC PH101, Tables 4–6 for MCC PH102, and Tables 7–9 for MCC CP102. A complete dataset containing all 614 sets of raw data are given in the supplement file.

## 2 Experimental Design, Materials, and Methods

Three types of microcrystalline cellulose (MCC) were considered, including MCC Avicel PH101 and PH102 (FMC; Biopolymer, Cork, Ireland) and Celospheres, referred to as MCC CP102 (CELPHERE® CP102; Asahi Kasei, Japan). The particle sizes (represented by median diameter, d50) are 88.08±0.03, 108.62±3.69 and 186.60±0.9  μm for MCC PH101, MCC PH102 and MCC CP102, respectively. The sphericities (represented by median sphericity, s50) of these three materials are 0.65±0.01, 0.71±0.01, 0.91±0.01, respectively 1.

A model rotary die filling system was developed and a detailed description of the system can be found in our recent research paper 2. When the filling system runs at different turret speeds and paddle speeds, the weight of the powder deposited into the four dies were different. At the end of each test, the powder in the die was weighed and the filling ratio in the die was calculated. Then, the results for the 4 dies and various operating conditions were analysed.

Three sets of experiments were performed for each material in order to understand the effect of process parameters on die filling: a) A controlled paddle speed of 50  rpm but with varying turret speeds; b) A controlled turret speed at a relatively high value but varying paddle speeds; c) A control turret speed at a relatively low value but varying paddle speeds. The turret speed was set in the range of 5 - 900  mm/s. At the speed of 5  mm/s, each material can fully fill in the die. Therefore, this speed was used to determine the maximum amount of powder in a fully filled die. The actual turret speed was monitored and calibrated using an Advent optical tachometer (A2103/LSR, Compact Instruments, UK). The paddle speed was set in the range of 10 - 100  rpm. To reach the specified speed, the acceleration was set to 50  Hz/ms.

**Table 1a:** Average fill ratios for MCC PH101 at a fixed paddle speed of 50 rpm.

Die No.	Turret speed (mm/s)	Average fill ratio
1	8.36	0.95
	24.69	0.66
	41.03	0.69
	57.36	0.49
	81.86	0.44
	163.53	0.40
	245.20	0.33
2	8.36	0.98
	24.69	0.89
	41.03	0.86
	57.36	0.79
	81.86	0.42
	163.53	0.41
	245.20	0.32
3	8.36	0.99
	24.69	0.88
	41.03	0.72
	57.36	0.54
	81.86	0.39
	163.53	0.31
	245.20	0.29
4	8.36	0.99
	24.69	0.98
	41.03	0.87
	57.36	0.95
	81.86	0.60
	163.53	0.54
	245.20	0.46

**Table 1b:** Mean fill ratios and the CV values for MCC PH101 at a fixed paddle speed of 50  rpm.

Turret speed (mm/s)	Mean fill ratio	CV(%)
8.36	0.98	2.12
24.69	0.85	15.90
41.03	0.79	12.15
57.36	0.69	31.18
81.86	0.46	20.11
163.53	0.41	22.42
245.20	0.35	22.14

**Table 2a:** Average fill ratios for MCC PH101 at a fixed turret speed of 24.69  mm/s.

Die No.	Paddle speed (rpm)	Average fill ratio
1	10	0.47
	30	0.48
	50	0.69
	70	0.59
	100	0.59
2	10	0.57
	30	0.74
	50	0.86
	70	0.54
	100	0.56
3	10	0.45
	30	0.57
	50	0.72
	70	0.58
	100	0.58
4	10	0.70
	30	0.91
	50	0.87
	70	0.97
	100	0.85

**Table 2b:** Mean fill ratios and the CV values for MCC PH101 at a fixed turret speed of 24.69  mm/s.

Paddle speed (rpm)	Mean Fill ratio	CV (%)
10	0.55	29.59
30	0.68	22.31
50	0.79	20.39
70	0.67	24.11
100	0.64	21.16

**Table 3a:** Average fill ratios for MCC PH101 at a fixed turret speed of 81.86  mm/s.

Die No.	Paddle speed (rpm)	Average fill ratio
1	10	0.30
	30	0.33
	50	0.44
	70	0.38
	100	0.38
2	10	0.30
	30	0.35
	50	0.42
	70	0.48
	100	0.53
3	10	0.27
	30	0.34
	50	0.39
	70	0.35
	100	0.32
4	10	0.30
	30	0.44
	50	0.60
	70	0.57
	100	0.48

**Table 3b:** Mean fill ratios and the CV values for MCC PH101 at a fixed turret speed of 81.86  mm/s.

Paddle speed (rpm)	Mean fill ratio	CV (%)
10	0.29	18.10
30	0.37	23.65
50	0.46	19.26
70	0.44	20.12
100	0.43	22.00

**Table 4a:** Average fill ratios for MCC PH102 at a fixed paddle speed of 50  rpm.

Die No.	Turret speed (mm/s)	Average fill ratio
1	8.36	0.99
	41.03	0.77
	81.86	0.52
	163.53	0.42
	245.20	0.39
	326.87	0.34
	408.54	0.31
2	8.36	1.01
	41.03	0.83
	81.86	0.54
	163.53	0.40
	245.20	0.36
	326.87	0.36
	408.54	0.30
3	8.36	1.04
	41.03	0.92
	81.86	0.58
	163.53	0.40
	245.20	0.40
	326.87	0.29
	408.54	0.28
4	8.36	1.03
	41.03	0.95
	81.86	0.72
	163.53	0.62
	245.20	0.52
	326.87	0.50
	408.54	0.49

**Table 4b:** Mean fill ratios and the CV values for MCC PH102 at a fixed paddle speed of 50  rpm.

Turret speed (mm/s)	Mean fill ratio	CV(%)
8.36	1.02	2.03
41.03	0.87	9.83
81.86	0.59	15.07
163.53	0.46	23.01
245.20	0.42	16.51
326.87	0.37	24.85
408.54	0.35	27.65

**Table 5a:** Average fill ratios for MCC PH102 at a fixed turret speed of 41.03  mm/s.

Die No.	Paddle speed (rpm)	Average fill ratio
1	10	0.64
	30	0.63
	50	0.77
	70	0.65
	100	0.78
2	10	0.71
	30	0.70
	50	0.83
	70	0.69
	100	0.87
3	10	0.92
	30	0.88
	50	0.92
	70	0.81
	100	0.88
4	10	0.95
	30	0.93
	50	0.95
	70	0.95
	100	0.97

**Table 5b:** Mean fill ratios and the CV values for MCC PH102 at a fixed turret speed of 41.03  mm/s.

Paddle speed (rpm)	Mean fill ratio	CV (%)
10	0.81	18.71
30	0.78	18.31
50	0.87	9.83
70	0.78	17.53
100	0.88	8.90

**Table 6a:** Average fill ratios for MCC PH102 at a fixed turret speed of 245.2  mm/s.

Die No.	Paddle speed (rpm)	Average fill ratio
1	10	0.36
	30	0.38
	50	0.39
	70	0.36
	100	0.38
2	10	0.32
	30	0.34
	50	0.36
	70	0.32
	100	0.32
3	10	0.29
	30	0.36
	50	0.40
	70	0.35
	100	0.38
4	10	0.37
	30	0.41
	50	0.52
	70	0.39
	100	0.42

**Table 6b:** Mean fill ratios and the CV values for MCC PH102 at a fixed turret speed of 245.2  mm/s.

Paddle speed (rpm)	Mean fill ratio	CV (%)
10	0.33	10.60
30	0.37	7.80
50	0.42	16.51
70	0.35	8.06
100	0.38	10.79

**Table 7a:** Average fill ratios for MCC CP102 at a fixed paddle speed of 50  rpm.

Die No.	Turret speed (mm/s)	Average fill ratio
1	81.86	0.98
	245.20	0.98
	408.54	0.82
	490.21	0.74
	571.88	0.70
	653.55	0.64
	735.21	0.56
2	81.86	0.99
	245.20	0.99
	408.54	0.89
	490.21	0.81
	571.88	0.72
	653.55	0.64
	735.21	0.57
3	81.86	0.99
	245.20	0.98
	408.54	0.84
	490.21	0.77
	571.88	0.68
	653.55	0.62
	735.21	0.54
4	81.86	0.98
	245.20	0.98
	408.54	0.96
	490.21	0.83
	571.88	0.80
	653.55	0.71
	735.21	0.63

**Table 7b:** Mean fill ratios and the CV values for MCC CP102 at a fixed paddle speed of 50  rpm.

Turret speed (mm/s)	Mean fill ratio	CV(%)
81.86	0.99	0.74
245.20	0.98	0.52
408.54	0.88	7.34
490.21	0.79	5.03
571.88	0.73	7.26
653.55	0.65	6.02
735.21	0.57	6.38

**Table 8a:** Average fill ratio for MCC CP102 at a fixed turret speed of 408.54  mm/s.

Die No.	Paddle speed (rpm)	Average fill ratio
1	10	0.77
	30	0.81
	50	0.82
	70	0.87
	100	0.93
2	10	0.81
	30	0.84
	50	0.89
	70	0.89
	100	0.91
3	10	0.81
	30	0.86
	50	0.84
	70	0.89
	100	0.93
4	10	0.91
	30	0.95
	50	0.96
	70	0.97
	100	0.97

**Table 8b:** Mean fill ratios and the CV values for MCC CP102 at a turret speed of 408.54  mm/s.

Paddle speed (rpm)	Mean fill ratio	CV (%)
10	0.82	7.64
30	0.86	6.67
50	0.88	7.33
70	0.90	4.88
100	0.94	2.34

**Table 9a:** Average fill ratio for MCC CP102 at various paddle speeds but a fixed turret speed of 653.55  mm/s.

Die No.	Paddle speed (rpm)	Average fill ratio
1	10	0.55
	30	0.59
	50	0.64
	70	0.63
	100	0.65
2	10	0.53
	30	0.62
	50	0.64
	70	0.62
	100	0.67
3	10	0.53
	30	0.56
	50	0.62
	70	0.61
	100	0.62
4	10	0.59
	30	0.67
	50	0.71
	70	0.70
	100	0.70

**Table 9b:** Mean fill ratios and the CV values for MCC CP102 at a turret speed of 653.55  mm/s.

Paddle speed (rpm)	Mean fill ratio	CV (%)
10	0.55	8.97
30	0.61	7.77
50	0.65	5.57
70	0.64	5.28
100	0.66	4.88

The efficiency of die filling was evaluated in terms of the fill ratio, δ, defined as

(1) $$\delta =\frac{m}{M}$$

where m is the mass of powder in the die at a given turret speed and paddle speed, and M is the mass of powder in a completely filled die.

The critical turret speed is the maximum turret speed to enable the die completely filled, which can be used to characterise powder flowability [3,4]. The fill ratio can be described using

(2) $$\delta ={\left(\frac{v_{c}}{v_{t}}\right)}^{n}\;\left(v_{t}\;>v_{c}\right)$$

where v_c_ is the critical turret speed (in this paper, the v_c_ is determined for a paddle speed of 50  rpm), v_t_ is the specified turret speed and n is a dimensionless parameter.

## Declaration of Competing Interest

The authors declare that they have no known competing financial interests or personal relationships which have, or could be perceived to have, influenced the work reported in this article.

## Appendix Supplementary materials

Image, application 1
